# Structural basis for water modulating RNA duplex formation in the CUG repeats of myotonic dystrophy type 1

**DOI:** 10.1016/j.jbc.2023.104864

**Published:** 2023-05-26

**Authors:** Shun-Ching Wang, Yi-Tsao Chen, Roshan Satange, Jhih-Wei Chu, Ming-Hon Hou

**Affiliations:** 1 Institute of Genomics and Bioinformatics, National Chung Hsing University, Taichung, Taiwan; 2 PhD. Program in Medical Biotechnology, National Chung Hsing University, Taichung, Taiwan; 3 Institute of Bioinformatics and Systems Biology, National Chiao Tung University, Hsinchu, Taiwan; 4 Department of Biological Science and Technology, National Chiao Tung University, Hsinchu, Taiwan; 5 Institute of Molecular Medicine and Bioengineering, National Chiao Tung University, Hsinchu, Taiwan; 6 Department of Life Sciences, National Chung Hsing University, Taichung, Taiwan

**Keywords:** CUG repeats, myotonic dystrophy type 1, RNA, U–U mismatch, X-ray crystallography

## Abstract

Secondary structures formed by expanded CUG RNA are involved in the pathobiology of myotonic dystrophy type 1. Understanding the molecular basis of toxic RNA structures can provide insights into the mechanism of disease pathogenesis and accelerate the drug discovery process. Here, we report the crystal structure of CUG repeat RNA containing three U–U mismatches between C–G and G–C base pairs. The CUG RNA crystallizes as an A-form duplex, with the first and third U–U mismatches adopting a water-mediated asymmetric mirror isoform geometry. We found for the first time that a symmetric, water-bridged U-H_2_O-U mismatch is well tolerated within the CUG RNA duplex, which was previously suspected but not observed. The new water-bridged U–U mismatch resulted in high base-pair opening and single-sided cross-strand stacking interactions, which in turn dominate the CUG RNA structure. Furthermore, we performed molecular dynamics simulations that complemented the structural findings and proposed that the first and third U–U mismatches are interchangeable conformations, while the central water-bridged U–U mismatch represents an intermediate state that modulates the RNA duplex conformation. Collectively, the new structural features provided in this work are important for understanding the recognition of U–U mismatches in CUG repeats by external ligands such as proteins or small molecules.

The polymorphic nature of RNA is important for its diverse functions. RNA can undergo various motions and transitions, and presence of such heterogeneous conformations has important implications for ligand binding, signaling, or catalysis (1, 2, 3). The key features influencing RNA structures involve specific base pairing and solvent-mediated interactions (4, 5, 6), which are central to the biological functions and recognition by external ligands. A generally accepted signature of a base pair is two direct hydrogen bonds. However, studies have shown that base pairs in RNA structures are even connected by one or no direct hydrogen bonds (7, 8, 9). The stability of such base pairs is likely provided by the stacking interactions with neighboring bases as well as by water-mediated base pairing, since many structural studies have found hydrogen-bonded water between the paired bases in RNA (10, 11, 12). For example, in myotonic dystrophy type 1 (DM1)-related CUG repeat RNA structures, conserved water molecules in U–U mismatches are frequently observed in the major and minor grooves (13).

The structural properties of CUG repeat RNA have been studied with a range of experimental and theoretical methods, including NMR, X-ray crystallography, single-molecule techniques, and molecular dynamics (MD) simulation (14, 15, 16). The crystal structure of an A-form RNA duplex containing two CUG repeats, in particular, showed an asymmetrically inclined "stretched U–U wobble" conformation (13). Such asymmetric U–U mismatches are commonly observed in CUG repeat RNA structures, and the base pairs therein exhibit different numbers of hydrogen bonds ranging from zero to one or two. These results imply that the U–U mismatches likely adopt several different conformations (17). For example, in addition to the predominant asymmetric conformations, the presence of a symmetric U–U mismatch pairing in CUG RNA duplex has been proposed (18). Despite the extensive structural characterization of CUG repeats, atomic-scale information regarding the structural transition between RNA conformations, particularly the role of water, has been difficult to acquire. A better understanding of conformational changes in base pairs, base stacking, and solvent-mediated interactions is important to uncover how RNA repeats are involved in disease pathogenesis by the mismatch structures.

To provide the key information of water mediation within pathogenic RNA structures, we solved an RNA crystal structure containing three continuous CUG repeats and identified three different states of U–U mismatches, MM1, MM2, and MMT. The first and third mismatches exhibit a mirror isoform, asymmetric, single hydrogen bonded geometry in which one of the uridines is tilted into the minor groove (MM1 and MM2 states). At the central repeat, we discover a new type of U–U mismatch displaying a planar symmetry with two uridines separated about 6 Å apart (MMT state). The MMT state contains a central water molecule bridging the two uridines, resulting in a symmetric duplex structure. The detailed structural analysis revealed how a specific U–U mismatch conformation can modulate the CUG-repeat RNA features. We also used all-atom MD simulation in explicit solvent to understand the exchange between the different conformations of U–U mismatch in the CUG RNA structures. Our analysis indicates that MM1 and MM2 are the predominant structural forms, while MMT is an intermediate conformation. The presence of U–U mismatches is shown to disrupt adjacent G–C base pair stacking interactions in a manner that depends on the mismatch conformation. These results provide new structural insights into the U–U mismatches in DM1-related CUG repeat RNA, which are unique features for recognition by CUG-binding proteins or small molecule ligands.

## Results

### Crystal structures of CUG repeat RNA exhibit symmetric A-form-like duplex

To reveal the structural impact of U–U mismatches in CUG repeats, we determined the crystal structure of a 13-mer RNA nucleotide consisting of three continuous CUG repeats in a trigonal space group *P*3_2_21. The crystal diffracted X-rays at a resolution of 1.8 Å. A clear electron density map resolved for all RNA heavy atoms in the final refinement indicates no disorder in the structure (Fig. S1*A*). The biological assembly formed an intermolecular duplex in the crystal, while the asymmetric unit contains only a single RNA strand, indicating a 2-fold crystallographic symmetry. This RNA is denoted as M3 duplex in the following discussion. We also solved another crystal structure containing two U–U mismatches and a A–U Watson–Crick pair in the center at a resolution of 1.58 Å that similarly shows a duplex in the asymmetric unit and is referred to as M2 duplex (Fig. S1*B*). The five U–U mismatches from two crystal structures showed a high-quality fit of the electron density map for all atoms and water molecules mediating these mismatches (Fig. S2). The detailed crystallographic and final refinement statistics of the structures are given in Table 1.

**Table 1:** Crystallographic and refinement statistics of two RNA duplexes, M2 and M3, presented in this study

Data collection statistics	M2 duplex	M3 duplex
Sequence	r(UUCUGCUGCUGAA/UUCUGCAGCUGAA)	r(UUCUGCUGCUGAA)_2_
Beamline	NSRRC, BL15A1	NSRRC, TPS 05A
Detector type	RAYONIX MX300HE	RAYONIX MX300-HS
Wavelength [Å]	1.0000	1.0000
Data collection temperature [K]	100	100
Space group	*P*3_2_	*P*3_2_21
Cell dimensions		
a, b, c [Å]	49.22, 49.22, 35.80	49.26, 46.26, 36.14
α, β, γ [°]	90, 90, 120	90, 90, 120
Resolution range [Å]^a^	30.00–1.58 (1.58–1.64)	30.00–1.87 (1.87–1.94)
Total reflections	126,315	44,478
Unique reflections	12,483	4355
Completeness [%]^a^	94.0 (100)	99.4 (100)
Mean I/σ [I]^a^	30.86 (7.01)	30.85 (7.20)
R-merge [%]^a^	0.049 (0.398)	0.059 (0.383)
Redundancy^a^	10.8	10.1
Refinement statistics		
R-work/R-free	0.23/0.25	0.21/0.22
No. of nonsolvent atoms	550	274
No. of solvent atoms	113	58
Average B-factor [Å^2^]	28.2	30.0
R.m.s.d. bond lengths [Å]	0.004	0.004
R.m.s.d. bond angles [°]	0.89	0.85
PDB code	7Y2P	7Y2B

a Outer shell statistics are shown in parenthesis.

**Figure 1:**
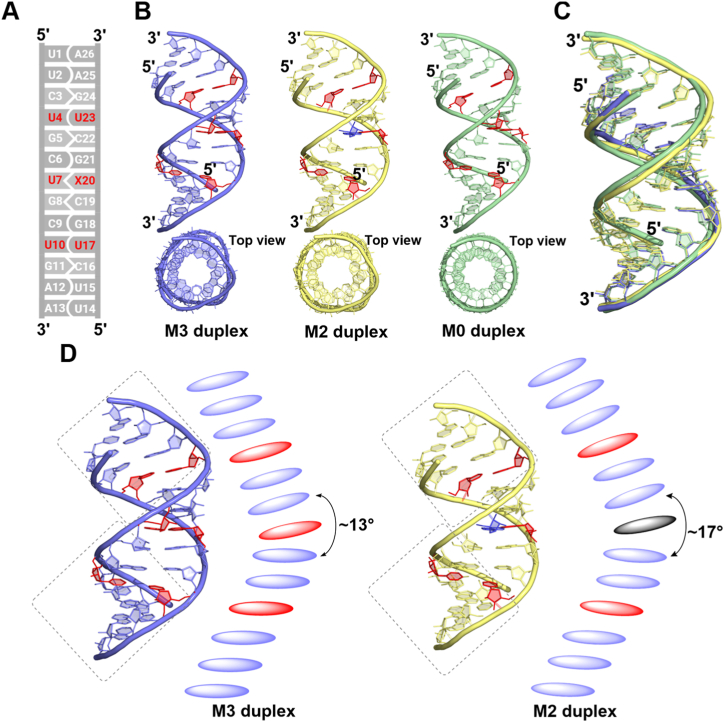
**Overall structural assembly of CUG repeat RNA structures.***A*, schematic representation of the CUG repeat sequences presented in this work. The bases are numbered from 1 to 13 in one strand while the complementary strand bases are numbered from 14 to 26. U–U mismatches are highlighted in red. X20 denotes either uracil (U) or adenine (*A*) nucleobase. Biological assembly of (*B*) M3 RNA and M2 RNA duplex structures. M0 duplex represents a typical A-form RNA modeled by using Discovery studio client package v19.1 for comparison. *C*, superimposition of M3, M2, and a typical A-form RNA, M0 duplex, is shown. *D*, the bending at the central CUG repeat is shown for M3 (*left*) and M2 (*right*) structures.

The oligonucleotides in these duplexes are numbered from U1 to A13 in one strand and U14 to A26 in the other complementary strand in the 5'→3′ direction (Fig. 1*A*). The two RNA duplexes adopt an A-form as based on the C3′-*endo* conformation of the sugar pucker and average glycosyl torsion angles (χ) of about −160° for all residues (Fig. 1*B*). The averaged helical twist (31.6°) and rise (2.7 Å) in both M2 and M3 are close to those in A-form structures. The two RNA duplexes exhibit end-to-end stacking to generate infinite pseudocontinuous CUG helices, which resemble the stem structure of stem-loops known to form in long CUG repeats (Fig. S3). For comparison, we built a uniform model of ideal A-form RNA with same sequence using Discovery studio client package v19.1 that is referred to as M0 duplex. The all-atom root mean square (r.m.s.) deviation of the ideal A-form RNA duplex to the M3 and M2 RNA duplexes is 0.8 and 1.1 Å, respectively, while the r.m.s. deviation between M3 and M2 structures is 0.2 Å (Fig. 1*C*). The sum of roll angles at the central base pair steps of the M3 and M2 duplexes is 13° and 17°, respectively, indicating a sharp bending of the RNA toward the major groove (Fig. 1*D*). Interestingly, the helical twist at the three U–U mismatch sites in the M3 duplex has larger values (average = 26.3 ± 1.0°) compared with those in M2 (average = 21.1 ± 0.5°). Local differences in translational parameters are also observed in both duplexes. The upper half of both RNA duplexes shows positive values for base pair buckle, while the lower half shows negative buckle values. The C–G and G–C base pairs adjacent to the U–U mismatches in the M3 RNA showed excessively high buckle values (up to ± 11.6°) compared with the M2 duplex (buckle values between 6 and -7°) (Fig. S4). In contrast to the previously reported structures of CUG RNA, where RNA usually forms an asymmetric duplex, an unprecedented, symmetric A-form duplex conformation is identified in the M3 structure of three continuous CUG repeats.

### Distinct U–U mismatch geometries in the crystal structures of CUG repeat RNA

**Figure 2:**
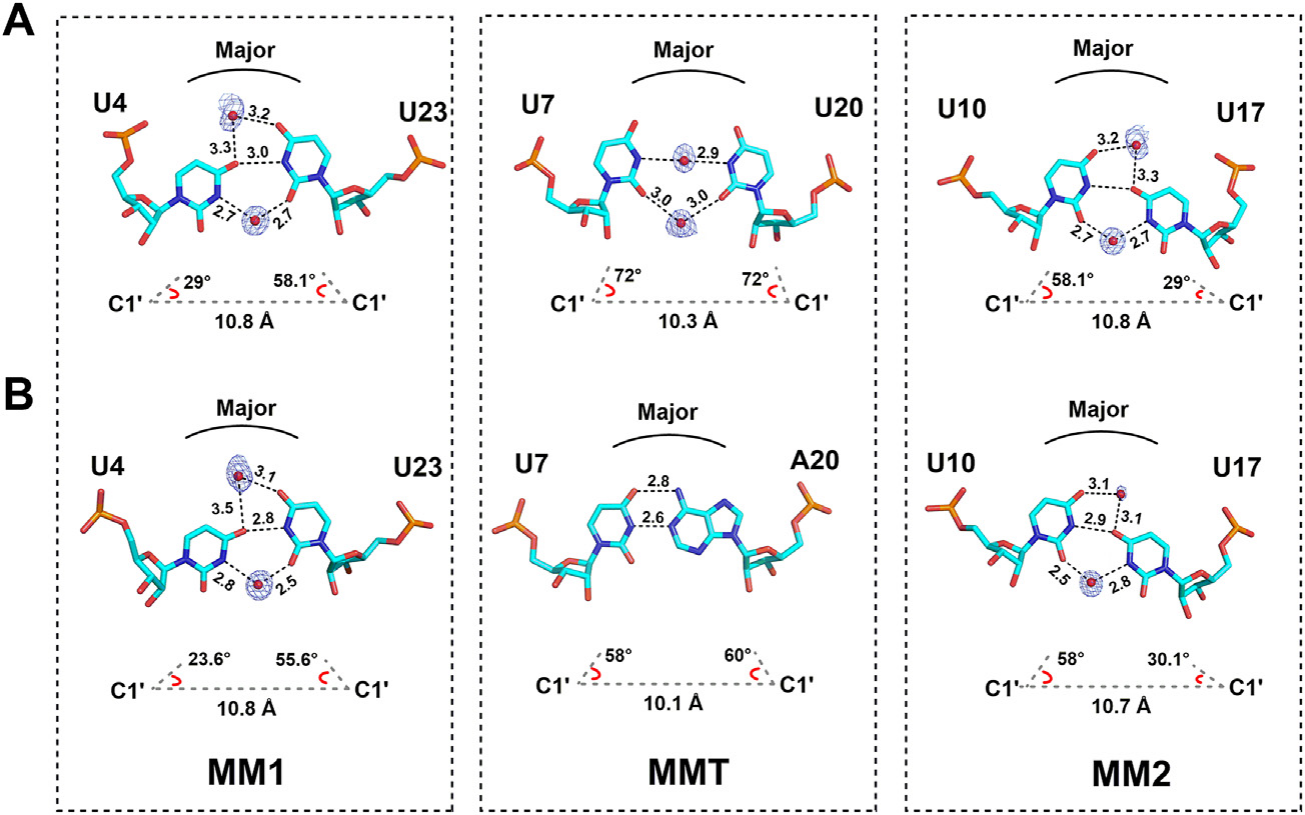
**U-U mismatch geometries in the crystal structures.** Three different states of U–U mismatch geometries are observed in the *A,* M3 and *B,* M2 RNA crystal structures. The MM1 and MM2 states showed inclined asymmetric mismatch pair formation stabilized by one direct hydrogen-bonded interaction. In the MMT state, the central U–U mismatch pair does not show any direct H-bonded interaction while the central A–U base pair is stabilized by two hydrogen-bonded interactions. Water molecules (*red spheres*) bridging U–U mismatches in the major and minor grooves are shown with the refined 2mF_o_–DF_c_ maximum likelihood-weighted Fourier electron density map countered at the 1.0 σ level. The values for inclination angles (λ angle) and C1′–C1′ distances are given below each mismatch type.

The two crystal structures presented here have five U–U mismatches stacked between C–G and G–C Watson–Crick base pairs. To get insight into the polymorphic nature of U–U mismatch conformations in these sequences, we compared the geometries of these mismatches. The majority of the mismatches are inclined toward the minor groove and shifted away from the helical axis, and three mismatch types as MM1, MM2, and MMT are identified (Fig. 2). The inclination angle (λ) between the glycosidic bond and the line connecting the two C1′–C1′ atoms in a base pair was used to quantify the degree of inclination of the nucleic acid base pairs (19). The first U4–U23 (MM1) and the third U10–U17 (MM2) mispair form only a single hydrogen bond between the O4 oxygen atom of U4 (U17) and the N3 amino atom of the complementary U23 (U10) nucleotides. The U–U mismatches show negative base pair opening (about −23° to 27°), indicating that they remain within the helix and hydrogen-bonding interaction can still be formed. The U–U mismatches are staggered such that the hydrogen bond–accepting residue is inclined into the minor groove, while the complementary uridine is displaced toward the major groove. In the M3 RNA structure, this staggering results in a λ-angle of about 29° for the inclined uridine, while the complementary residue has a λ-value of about 58.1° (Fig. 2*A*). In the M2 crystal, the λ-angle showed more variations with lower values of 26.8 ± 4.5° and 56.7 ± 1.5°, respectively, indicating that these U–U mismatches are more inclined toward the minor groove compared with those in the M3 structure (Fig. 2*B*). The average C1′–C1′ interstrand distance measured between these U–U mismatches is 10.8 ± 0.1 Å, which is higher than that of the standard A-helix (10.4 Å).

On the other hand, the central U7–U20 mismatch in the M3 crystal structure shows a unique symmetrical conformation with no inclination of the two uridines (MMT type). The values for the λ-angle are about 72° for both uridine bases within the mismatch. These λ-angle are normally observed for Watson-Crick base pairs (left and right λ-angles around 54°) of the A-form helix. Therefore, upon replacing the central U7-U20 mismatch with U7-A20 Watson-Crick base pair, the λ angles are about 58° and 59.9°, respectively. The U7-U20 mismatch shows a shear and buckle value of zero, compared to −0.2 Å and 0.4 Å in the U7-A20 base pair, respectively (Fig. S5*A*). Moreover, the base pair opening is about 35°, resulting in a large distance between the carbonyl oxygen and the N3 amino group of the two uridine residues, so there is no direct hydrogen bonding (Fig. S5*B*). In contrast, the U7–A20 formed two hydrogen bonds with a lower base pair opening of about 6°. Based on the analysis of nine CUG repeats within a single crystal structure, Tamjar *et al.*(18) proposed that about 15% of U–U mismatches would adopt symmetric conformations in CUG repeats without hydrogen bonds. As a critical assessment of this proposition, our M3 crystal structure shows that a perfectly symmetrical U–U mismatch was formed in the RNA structure of three continuous CUG repeats.

### Specific solvation structures correlate with different U–U mismatch geometry in CUG repeat RNA

The presence of water molecules in the minor and major grooves forms hydrogen-bonding interactions with the U–U mismatches. In both the crystal structures presented here, each U–U mismatch is solvated by two water molecules, one in the minor groove and the other in the major groove. In the U4–U23 and U10–U17 mismatches of the M3 duplex, the O2 oxygen and the N3 amino nitrogen form hydrogen bonds with a common water in the minor groove and the distance to water oxygen is 2.7 Å (Fig. 2*A*). On the other hand, the two O4 carbonyl atoms of the U4–U23 and U10–U17 mismatches form hydrogen bonds with the common water at 3.2 to 3.3 Å in the major groove (Fig. 2*A*). In the M2 duplex, the hydrogen bonds of U4–U23 and U10–U17 mismatches with the water molecules are 2.5 Å and 2.8 Å in the minor groove and 3.1 Å and 3.5 Å in the major groove, respectively (Fig. 2*B*). In the M3 structure, the U7–U20 mismatch pair showed a unique planar water-bridged interaction. The symmetrically stretched U–U form hydrogen bonds between two carbonyl oxygen atoms O2, each 3.0 Å apart. Another water bridges the interactions between two N3 amino atoms of two uridine residues with a short intermolecular distance of 2.9 Å. We named the current base pairing as a water-bridged U-H_2_O-U mismatch pairing. The current crystal structure is the first to capture such unique geometry in U–U mismatches, highlighting the importance of water molecules in mediating nucleic acid structural motifs.

**Figure 3:**
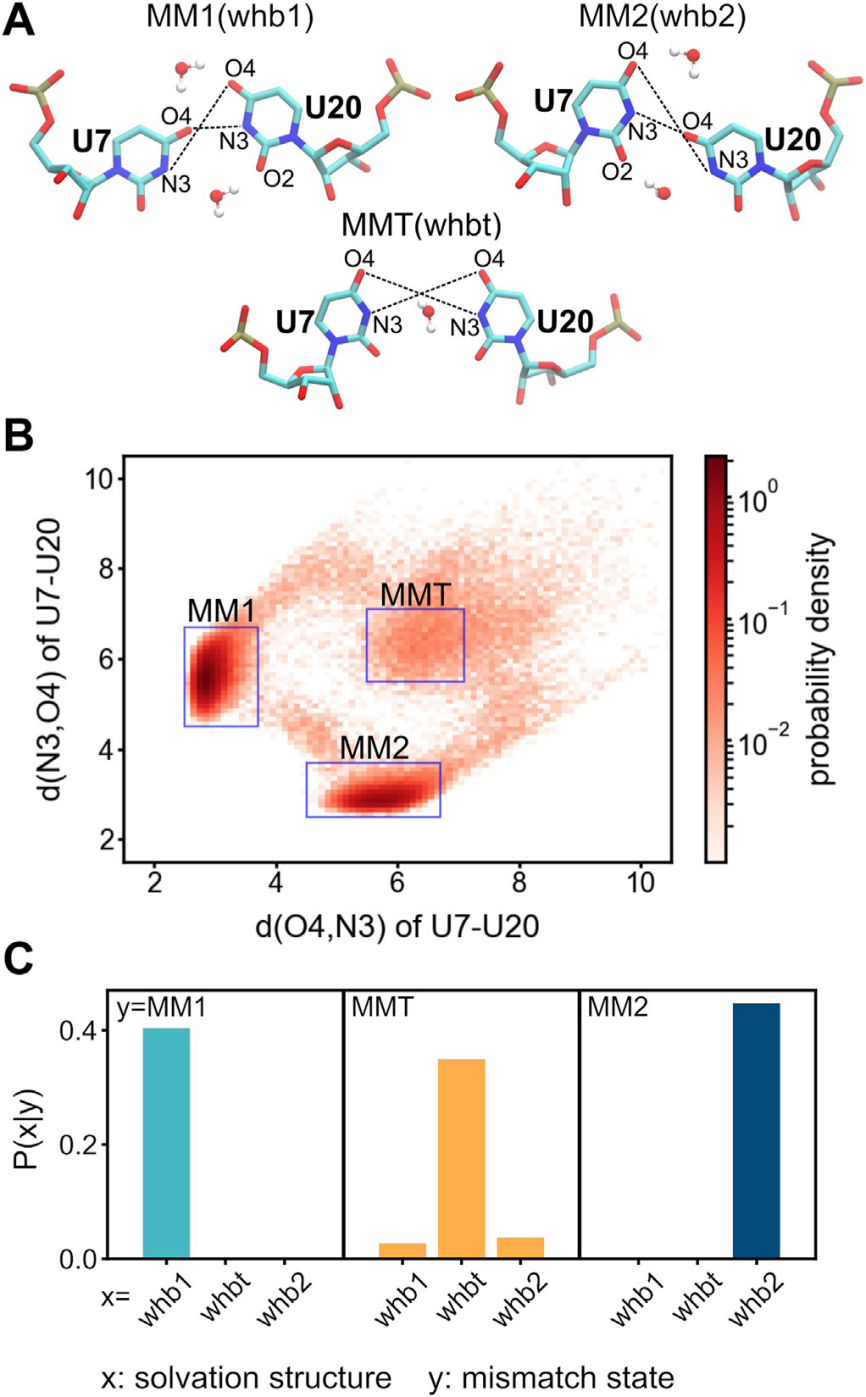
**The three distinct states of U–U mismatch and their corresponding solvation structures.** Here, the central U–U mismatch of M3 duplex, U7–U20, is used to present the findings observed in the molecular dynamics simulations. *A*, two order parameters, the distance between O4 atom in U7 and N3 atom in U20 (d(O4,N3)) and the distance between N3 atom in U7 and O4 atom in U20 (d(N3,O4)), are combined as the indicator of the state of U–U mismatch. For the solvation structures whb1 and whb2, the bridging water molecules in the major-groove side are those within 3.5 Å from two O4 atoms and the bridging water molecules in the minor-groove side are those within 3.5 Å from N3 atom and O2 atom. For the solvation structure whbt, the bridging water molecules in the middle of U–U mismatch are those within 3.5 Å from two N3 atoms. *B*, the joint probability density of d(O4,N3) and d(N3,O4). The definitions of MM1: 2.5 ≤ d(O4,N3) ≤ 3.7 and 4.5 ≤ d(N3,O4) ≤ 6.7, MM2: 4.5 ≤ d(O4,N3) ≤ 6.7 and 2.5 ≤ d(N3,O4) ≤ 3.7, and MMT: 5.5 ≤ d(O4,N3) ≤ 7.1 and 5.5 ≤ d(N3,O4) ≤ 7.1. *C*, the probability of finding a specific solvation structure x given a particular U–U mismatch state y.

Thus, the crystal structures of CUG-repeat RNA exhibit three distinct MM1, MM2, and MMT forms of U–U mismatches bridged specifically by water molecules. In order to further quantify the distribution of these three states, we performed 1-μs MD simulations starting from the M2 and M3 duplexes in explicit solvent model. The results of the central U–U mismatch in M3 duplex, U7–U20, are presented in Figure 3, while the qualitatively similar data of the other U–U mismatches can be found in Figs. S6 and S7. The distance between atom O4 of U7 and atom N3 of U20 and the distance between atom N3 of U7 and atom O4 of U20 were used to designate the U–U mismatch state (Fig. 3*A*). The profile of joint probability density of d(O4,N3) and d(N3,O4) was evaluated based on the 1-μs trajectory data (Fig. 3*B*). It can be observed that MM1 and MM2 are the two major forms of U–U mismatch, and MMT has a minor population. Furthermore, the time series of d(O4,N3) and d(N3,O4) indicate that transition between MM1 and MM2 occurs frequently during the dynamical simulation, and MMT is an intermediate state between the two dominant structural forms (top panel of Fig. S8*C*). Similar to the crystal structure, we also observed three distinct types of solvation structures, whb1, whb2, and whbt, during the simulations. Interestingly, the probability of finding the whb1, whb2, or whbt solvation state is shown to depend on the particular U–U mismatch state (MM1, MM2, MMT) (Fig. 3*C*). For example, whb1 is mostly found in the MM1 state while whb2 is specific to MM2. For MMT, in particular, the identified solvation structure tends to be whbt. Such dynamics and solvation structures of the U–U mismatch state are illustrated in Movie S1 (MM1 to MM2) and Movie S2 (MM2 to MM1). These results indicate that the distinct structural states of U–U mismatch would imprint recognizable patterns in the surrounding solvation environments.

### Single-sided cross-strand stacking interactions of U–U mismatches dominate CUG repeat structure

**Figure 4:**
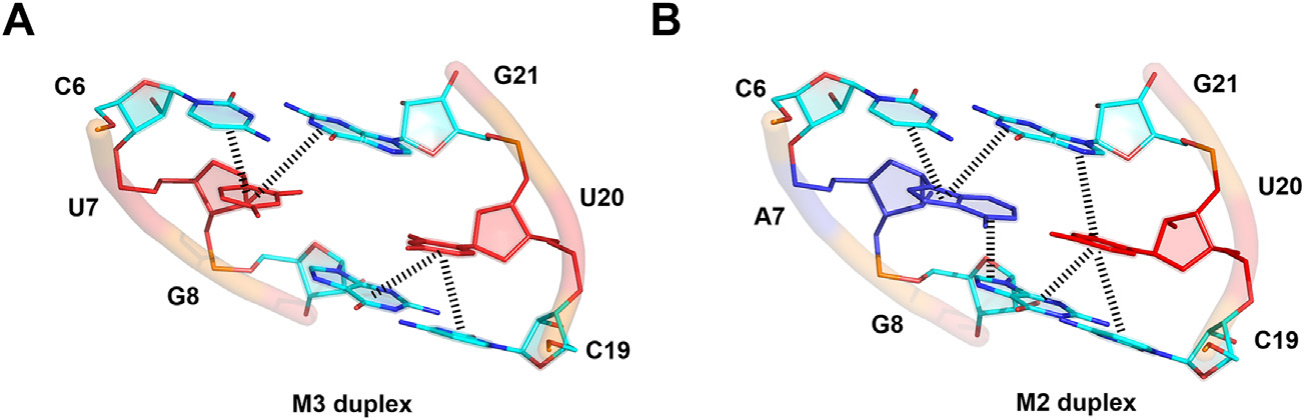
**Stacking interactions observed in the CUG-repeat RNA crystal structures.***A*, the M3 duplex exhibits single-sided cross-strand stacking interactions with adjacent C–G and G–C base pairs. *B*, replacing the U–U mismatch with A–U base pair in M2 duplex shows stacking interactions on either side of the adjacent base pairs.

Stacking interactions play an important role in maintaining nucleic acid structures (20, 21). Depending on the type and inclination of the uridines, the CUG repeats reveal different types of stacking interactions. In the current crystal structures, the uridine bases at the first U4–U23 and the third U10–U17 mismatch sites show intrastrand stacking with the five-membered ring of the adjacent guanosine on either side. The uridines also show a slight level of cross-strand stacking with the six-membered ring of the guanosine in the opposite strand. The stacking of the uridines with cytosine base depends on uridine geometry. The uridine inclined toward the minor groove does not stack with the adjacent cytosine, whereas the complementary uridine shows stacking interactions with the adjacent cytosine on the same strand. In the M3 structures, the central symmetric U–U mismatch shows a high stretch and high opening angle, which pushes two uridines away from each other. This particular situation resulted in a single-sided cross-strand stacking of one of the uridines (U7) with C6–G21, while another uridine (U20) is stacked with base pair C19–G8 (Fig. 4*A*). In contrast, when the central U–U mismatch is replaced with A–U Watson–Crick base pair, the lower base-pair opening retained the base pair within a helical axis and formed continuous stacking interactions on both sides of the A–U pair, similar to a standard A-form duplex (Fig. 4*B*).

Consistent with the structural findings, the MD simulation results also exhibit the dependence on the different structural form of U–U mismatch and cross-strand stacking interactions. For example, the U7→G21 cross-strand stacking (where the → notation indicates the stacking interaction) appears to go with U7–U20 being in the MM1 state (Movie S3). On the other hand, the U20→G8 cross-strand stacking tends to occur when the U7–U20 mismatch pair takes the MM2 form (Movie S4). Interestingly, when U7–U20 is in the intermediate MMT state, the bridging water molecule in the middle of U7–U20 pushes the two uridines outward, causing the canonical stacking interactions U7→C6 and U20→C19 to be well maintained (Movie S4). The details of correlation between cross-strand stacking and the U–U mismatch structures can be found in the supplementary note (Figs. S8 and S9).

## Discussion

Aberrant expansion of trinucleotide repeats is a major cause of many neurodegenerative diseases (22, 23). Of these repeats, CUG repeat RNA expansion in the 3′-untranslated region of dystrophia myotonica protein kinase mRNA has been shown to play an important role in the pathogenesis of DM1 (24, 25). The expanded CUG RNA forms a hairpin structure and binds the muscleblind like splicing regulator 1 (MBNL1) protein, resulting in splicing defects in the insulin receptor and muscle main chloride ion-related pre-mRNAs (26, 27). Characterization of such RNA structures involved in the pathogenicity of DM1 is important both for understanding the disease mechanism and for drug development (28, 29). Indeed, the crystal structures of CUG RNA repeats with different repeat lengths have been determined by many groups (9, 13, 18, 30, 31). These structures adopt the A-form conformation and represent the stem region of the “stem loop” normally found in the expanded CUG RNA repeats. The stability of these structures is inferred from the C–G and G–C base pairs flanking a central U–U mismatch. The U–U mismatch pairs are usually flexible and exhibit a variety of conformations with a different number of hydrogen bonds. Interestingly, MBNL showed high binding affinity to U–U mismatch containing short CUG repeat sequences suggesting that the local variations in short CUG stretches are important determinants of DM1 pathogenesis (32). Because of their conformational flexibility, it is assumed that the MBNL1 protein can recognize CUG repeats *via* an induced fit mechanism. Since no complex structure of MBNL1 bound to CUG repeats is available, most studies largely depend on RNA features in understanding the binding mechanism of MBNL. Thus, the availability of new crystal structures could shed light on this important pathogenic interaction.

To explore the U–U mismatch polymorphism and its implications for CUG RNA, we determine crystal structures containing successive CUG repeat motifs. Consistent to previous observations, the current CUG RNA adopts an A-like conformation with different U–U mismatch geometries. In this study, we particularly focused on using three consecutive CUG repeats as these repeat structures correspond closely with structures of longer CUG repeat duplexes. In the context of other sequences, U–U mismatches led to shorter C1′–C1′ distances with large values of the inclination angle λ, resulting in chemical asymmetry (33). In the CUG context, however, an unusually large distance between two uridines resulted in a "stretched" U–U geometry (13). Based on the uridine tilt and the number of hydrogen bonds between two uridines, Coonrod *et al.*(31) classified the U–U mismatches into six different types. Of the five U–U mismatches resolved in this study, two U–U mismatches follow type II (MM1 state) and type IV (MM2 state), with a single hydrogen bond but different λ-angles. The central U–U mismatch pairs adopt a completely new symmetric geometry without direct hydrogen bonding. Instead, this mismatch pair is mediated by a water molecule anchored between the N3 atoms of the two uridine bases. Around each U–U mismatch, specific solvation patterns are identified in the major groove and in the minor groove that appear to compensate the hydrogen bonding of the unpaired uridines. However, due to the tilting of one of the uridines, the bridging water molecule usually remains outside the mismatch pair. A unique symmetric U–U mismatch geometry is identified in this work and exemplifies that a solvent molecule can bridge the two N3 atoms of uridines. A consequence of this novel conformation is an environment that is readily accessible to external ligands and can therefore be a useful guide for molecular design of CUG-targeted small molecules. The dynamic behavior of U–U mismatches in CUG repeats was investigated by Yildirim *et al.*, González *et al.*, and Parkesh *et al.* using MD simulations and NMR (17, 34, 35). Their analyses suggested that U–U mismatches can form different conformations with four main possibilities, including zero, one, or two direct hydrogen bonds and water-mediated pairings. Here, we identified unprecedented correlation between the different states of U–U mismatches as well as the changes in the surrounding solvent molecules. For example, when the transition between MM1 ↔ MM2 states occurs, the solvation states are also interconverted between whb1 and whb2, whereas for the intermediate MMT state conformation, the solvation remained in the whbt state. As water plays a crucial role in protein–nucleic acid or drug–nucleic acid interactions networks (36, 37), the specific solvation states of U–U mismatches could be important determinant for protein or small molecule to bind CUG RNA repeats.

The structural data and MD analysis conducted here further delineate the crucial features of CUG RNA, which could be important for the recognition by CUG-binding ligands. For example, the structure of the MBNL1 zinc finger motif bound to r(CGCUGU) suggests that GC element recognition follows a chain-reversal loop trajectory (38). To form such reverse-topology, the base pairs within the secondary stem-loop structures of CUG RNA must be flexible. Our MD results suggested that the dynamic nature of the U–U mismatches leads to a disruption of the continuous stacking interactions at the 5′-CpU steps, causing the cytosine base to flip into the major groove of the duplex. Depending on the state of a U–U mismatch, cross-strand stacking with the adjacent C–G or G–C base pairs is shown to emerge. When the U–U mismatch is in the intermediate MMT state, stacking interactions with the bases on only one side were observed. These results suggest that the presence of a U–U mismatch would increase the overall flexibility of CUG RNA structures with reduced stability. To confirm these predictions, we performed CD spectral analysis and thermal stability assays for CUG RNA repeat duplex. Consistent with our structural and dynamical analysis, the CD spectra showed an A-form-like conformation with a negative band at 235 nm and a positive band at 275 nm, respectively. Compared with the spectra containing A–U Watson–Crick base pairing, the differences in CD intensity suggested higher levels of distortions in the CUG RNA duplex with lower stability (Fig. S10). These results suggest that the specific base pair structures and dynamics would provide fingerprint for recruiting cogent ligand proteins to interact with CUG RNA structures. Thus, our data underscore the importance of the polymorphic nature of U–U mismatch in DM1 pathogenesis.

**Figure 5:**
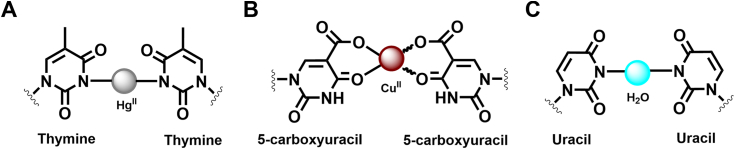
**Chemical representation of various metal-mediated base pairing.***A,* T-Hg^II^-T**.***B,* 5-caU-Cu^II^-5caU. *C,* the current U-H_2_O-U pairing.

Apart from the biological roles of novel U–U mismatches in CUG repeats, the naturally occurring nucleobases including thymine or cytosine selectively form stable metal-mediated base pairs. For example, Kondo *et al.* have shown that a mercury-mediated T–T mismatch is capable of causing a structural switch from a nonhelical form to the B-form of DNA (Fig. 5*A*) (39). A 5-carboxyuracil nucleobase has also been shown to form a copper-mediated pairing with various nucleobases, which has applications in the construction of diverse metallosupramolecules (Fig. 5*B*) (40). The current water-bridged U-H_2_O-U mismatch geometry observed in our crystal structure is similar to the metal-mediated pairs (Fig. 5*C*). Although we do not observe associated cations in the current crystal structure, the distance between two stretched uridines offers the possibility that the ordered water molecule could be replaced by metal ions. Such structural features could therefore provide a basis for rational design of metal-conjugated RNA nanomaterials containing U–U mismatches.

In summary, we have presented here new structural motifs of CUG repeat RNA duplexes with a water-bridged symmetric U–U mismatch geometry. Our results showed that the chemical symmetry of the central base pair is observed in U–U mismatches, suggesting that such U–U mismatch could exist as transient conformation in CUG repeat sequence context. More detailed studies would be required to confirm the biological consequences of symmetric U–U mismatch geometry. The new structural features presented here help to expand the current repertoire of U–U mismatches in CUG context. The structural understanding from this study would also help in the development of new U–U mismatch-selective chemical probes for targeting CUG repeats in DM1.

## Experimental procedures

### Synthesis of RNA oligonucleotides and sample preparation

RNA oligomers were commercially synthesized by MDBio, Inc and Genomics and purified by high-performance liquid chromatography. All chemicals used in this study were purchased from Sigma Chemical Co. All chemical solutions were prepared in 0.1% (v/v) diethyl pyrocarbonate-treated ddH_2_O. Oligonucleotides were dissolved in 0.1% (v/v) diethyl pyrocarbonate-treated ddH_2_O by heating at 95 °C for 5 min followed by slow cooling (−0.5 °C/min) to room temperature to allow annealing of duplexes. The absorbance of the oligonucleotide concentrations was determined using JASCO V-630 ultraviolet-visible spectrophotometer (JASCO International Co Ltd) with a quartz cuvette (1 cm path length) at a wavelength of 260 nm. The quantitative concentration of the oligonucleotides was calculated through Beer's law with the approximate values of extinction coefficients for each oligonucleotide (41).

### Crystallization

For the crystallization, HPLC-purified oligonucleotides were denatured by heating to 95 °C for 5 min and then stored on ice for annealing. To grow M3 RNA crystals, 0.2 mM RNA solution was mixed with mother liquid containing 50 mM 2-[4-(2-hydroxyethyl)piperazin-1-yl]ethane-1-sulfonic acid (pH 6.5), 3.5 mM spermine tetrahydrochloride, 10% (v/v) 2-methyl-2,4-pentanediol in 1: 1 ratio, and equilibrated with 300 μl mother liquid. Crystals suitable for X-ray crystallography experiments were obtained by the sitting-drop vapor diffusion method. After 1 to 2 weeks, translucent long rod-shaped crystals were obtained at 4 °C. To obtain M2 RNA crystals, 0.2 mM RNA oligonucleotides were mixed in solution containing 50 mM 2-[4-(2-hydroxyethyl)piperazin-1-yl]ethane-1-sulfonic acid (pH 6.5), 0.5 mM spermine tetrahydrochloride, 10% (v/v) 2-methyl-2,4-pentanediol in a 1: 1 ratio and equilibrated with 300 μl mother liquid. Bright and transparent broad rod-shaped crystals grew after about 1 week at 4 °C.

### Data collection, phasing, structure determination, and refinement

X-ray diffraction data were acquired using a fixed-exit double crystal monochromator with images collected on a Rayonix MX300HS CCD area detector and a Rayonix MX300HE CCD area detector, respectively, at the Biological Crystallography Facility of the National Synchrotron Radiation Research Center (NSRRC), Taiwan. Data collection wavelength of 1 Å was used for a single crystal cooled to 100K with a stream of nitrogen. Diffraction data reduction, processing, integration, and scaling were performed using the HKL-2000 package. PHENIX (version 1.18.2-3874) was used to determine the phases for M3 and M2 RNA duplexes using molecular replacement (Phaser MR) in space groups *P*3_2_21 and *P*3_2_, respectively. The crystallographic model of a typical A-form RNA duplex was created using Discovery Studio 2020 client software (version 20.1.0.19295) and used as template for initial phase determination of the duplexes. Structural refinement using phenix.refine in PHENIX (version 1.18.2-3874) (42) and WinCoot in CCP4i (version 0.8.9.2) (43) were performed. The final *2*F_*o*_–F_*c*_ electron density maps were generated using the fast Fourier transform in CCP4i and PyMOL (version 2.3.2), which was used to draw graphical representations of the refined structures.

### Calculation of structural parameters

A standard A-form RNA structure with the same sequence as CUG RNA duplex was created using Discovery Studio 2020 client software (version 20.1.0.19295) for structural comparison. The RNA structural parameters, including helix, torsion, local base pair parameters, and local base pair step parameters were analyzed using the Curves plus web server program (44). Values for base pair and base pair step parameters are provided in Tables S1 and S2. The calculation of the root mean square deviations (r.m.s.d.) and the crystallographic drawing were performed with PyMOL (version 2.3.2).

### Circular dichroism spectroscopy and thermal stability of RNA duplexes

Circular dichroism (CD) spectral analysis was performed at 25 °C on a ChirascanTM V100 CD spectrophotometer (version 4.8.3.313) with Pro-Data software suite (version 4.8.3.0) in quartz cuvettes with path length of 1 mm. RNA duplex, 10 μM, was mixed in a buffer containing 20 mM Mops (pH 6.5) and 1 mM spermine tetrahydrochloride. The samples were annealed in the buffer from 95 to 4 °C and then stored overnight. The determination of the ellipticity was carried out in the range of 400 to 200 nm, at a sampling rate [time per point (s)]. The ellipticity of the circular dichroism was superimposed and normalized by curve fitting using Pro-Data Software Suite core (version 4.8.3.0). To perform melting temperature (T_m_) analysis, RNA oligonucleotides were prepared in the same buffer (20 mM Mops [pH 6.5] and 1 mM spermine). Circular dichroism (medg) curves were determined by increasing the temperature from 4 to 95 °C at a rate of 1 °C/min and recording every 1 min at 268 nm.

### Molecular dynamics simulation

The crystal structures of M2 and M3 duplexes were used as the initial structures to perform MD simulations. Both systems were solvated in dodecahedron boxes of explicit water molecules with at least 10 Å between any nucleic atom and box edges. Na^+^ and Cl^−^ ions were added to achieve charge neutrality and ionic strength of 0.15 M. For the system of M2 duplex, there are 43 Na^+^ and 19 Cl^−^, and there are 44 Na^+^ and 20 Cl^−^ in the system of M3 duplex. The resulting M2 system has 20,898 atoms and the M3 system contains 21,326 atoms. The cut-off radius for van der Waals interactions and real-space particle-mesh Ewald terms of electrostatics (45) was 12 Å with a switching function effective at 10 Å. During the all-atom MD simulations, all bond lengths involving the hydrogen atom were constrained at the equilibrium values *via* LINCS (46). After initial minimization and 12-ns equilibration period, the production run of 1 μs was conducted at constant temperature (310 K) by Langevin dynamics and pressure (1.013 bar) by the Parrinello–Rahman barostat (47). All the equilibration and production runs were carried out using the GROMACS software (48) and the AMBER nucleic acid force field (49). A snapshot was saved every 10 ps during the production MD runs for distance analysis, thereby resulting in 100,000 structures for each system. All the distance analyses were carried out using the MDAnalysis package (50, 51).

## Data availability

The atomic coordinates and structure factors for the reported crystal structures have been deposited in the Protein Data Bank with accession codes 7Y2B and 7Y2P.

## Supporting information

This article contains supporting information.

## Conflict of interest

The authors declare that they have no conflicts of interest with the contents of this article.
