# Supplementary material for: Structural basis for water modulating RNA duplex formation in the CUG repeats of myotonic dystrophy type 1
Source: J Biol Chem. 2023 May 26;299(7):104864. doi: 10.1016/j.jbc.2023.104864 (PMC10316006; doi:10.1016/j.jbc.2023.104864)
Supplement: Supporting Data [file mmc5.pdf]

Supplementary Information For

Structural basis for water modulating RNA duplex formation in the CUG repeats of myotonic dystrophy type 1

Shun-Ching Wang[1,2,†], Yi-Tsao Chen[4,†], Roshan Satange[1,†], Jhih-Wei Chu[4,5,6,*], and Ming-Hon Hou[1,2,3,*]

[1]Institute of Genomics and Bioinformatics; National Chung Hsing University, Taichung 402, Taiwan.
[2]Ph.D. Program in Medical Biotechnology, National Chung Hsing University, Taichung 402, Taiwan.
[3]Department of Life Sciences, National Chung Hsing University, Taichung 402, Taiwan.
[4]Institute of Bioinformatics and Systems Biology, National Chiao Tung University, Hsinchu, 30068 Taiwan.
[5]Department of Biological Science and Technology, National Chiao Tung University, Hsinchu, 30068 Taiwan.
[6]Institute of Molecular Medicine and Bioengineering, National Chiao Tung University, Hsinchu, 30068 Taiwan.

*Corresponding Authors: Jhih-Wei Chu jwchu@nctu.edu.tw; Tel: +886 3 5712121 ext. 56996 and Ming-Hon Hou mhho@nchu.edu.tw; Tel: +886-4-2284-0338 (ext. 7011), Fax: +886-4-2285-9329

[†]Authors contributed equally to this work.

Contents:

Supplementary note 2
Supplementary figures 2
Supplementary tables...8
Supplementary movies 11

Supplementary note

Cross-strand stacking depends on the U-U mismatch structure

To understand the perturbations of U-U mismatch to the neighboring base pairings, the hydrogen bonding distances in the two adjacent C-G base pairs and the base-base distances along the same strand were calculated. Only the results of the central CUG in M3 duplex are presented in Figure S8 since the other U-U mismatches have qualitatively similar behaviors (Fig. S9). The time series of d(N4,O6) indicates that the U7-U20 mismatch in M3 would lead to base-pairing disruption in either C6-G21 or C19-G8. This alternative base-pairing disruption is found to correlate with the mismatch state of U7-U20. When U7-U20 was in MM1, base-pairing disruption tends to occur at C6-G21, while the disruption is more likely to be found at C19-G8 when U7-U20 is in MM2. Furthermore, cross-strand stacking is also observed in the MD simulations of U-U mismatch, and the green dotted lines in Figure S8A-B indicate the two major cases of G21-U7 and G8-U20. In the top panel of Figure S8C, the time series of the stacking distance, d(stacking), of C6-U7 (magenta), G21-U20 (orange) and G21-U7 (green), are shown to illustrate the cross-strand stacking. In particular, d(stacking) of G21-U7 or G8-U20 being shorter than 4 Å indicates cross-strand stacking. In the meantime, larger d(stacking) values of C6-U7 and G21-U20 show that the canonical stacking interactions in the A-form dsRNA are disrupted when the U-U mismatch and cross-strand stacking are present. Data for the other cross-strand stacking of G8-U20 are illustrated in the bottom panel of Figure S8C.

Supplementary figures

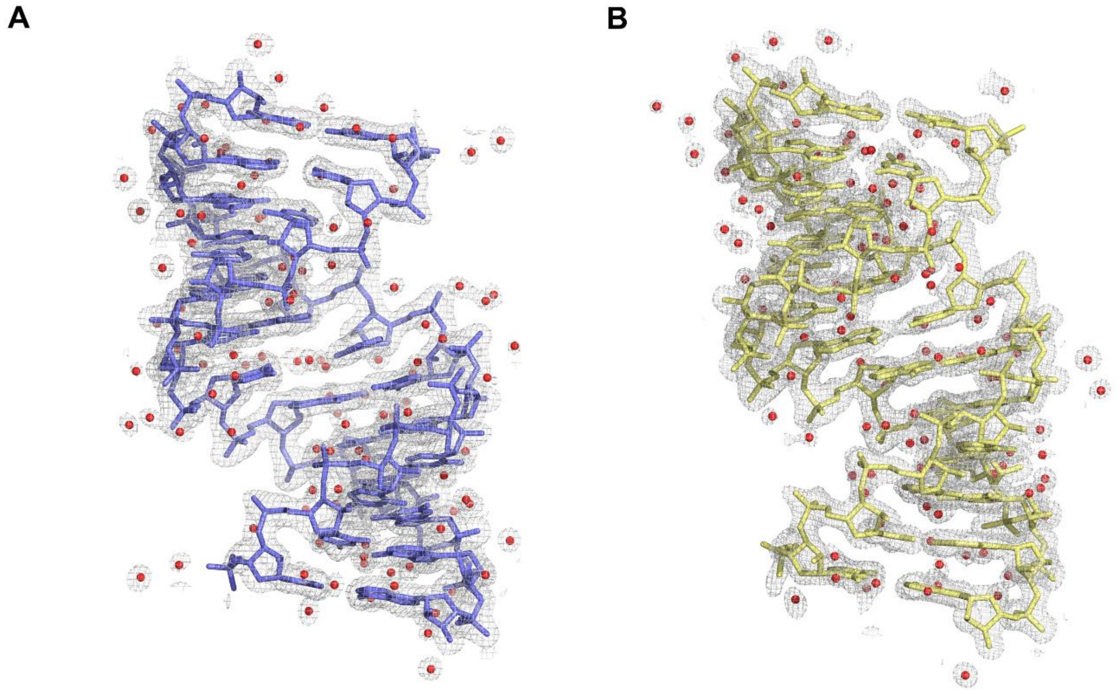

Figure S1: Overall crystal structures of **(A)** M3 and **(B)** M2 RNA duplexes showing $2F_o$-F_c electron density maps countered at 1.0 σ level.

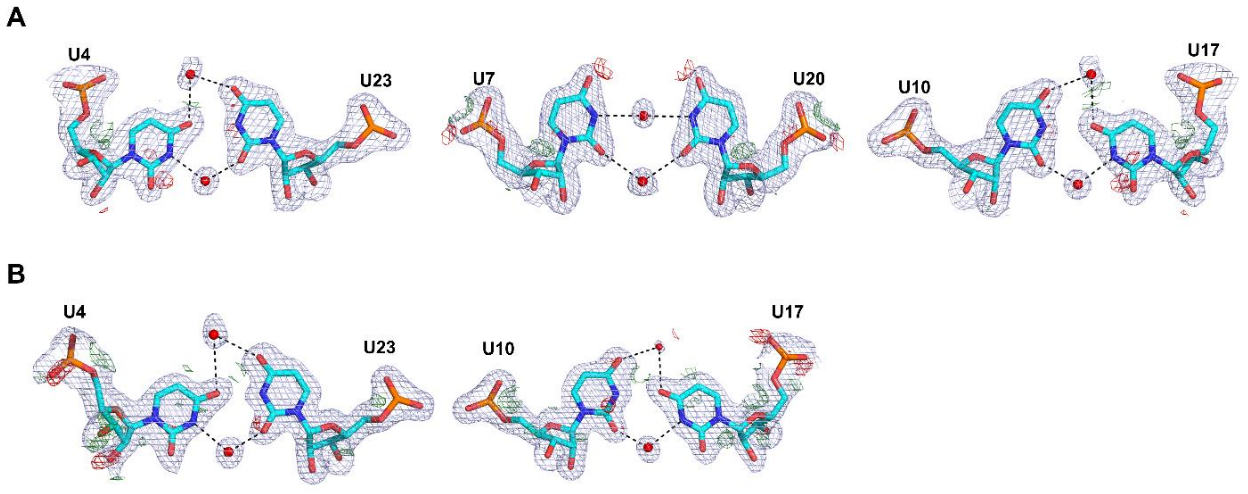

Figure S2: Correlation between electron density map quality and the atomic models of U-U mismatches in two crystal structures. The refined $2mF_o$-DF_c maximum likelihood-weighted Fourier electron density map in ccp4 format countered at the 1.0 σ level with a 1.6 Å carve radius (grey mesh) for five U-U mismatches in **(A)** M3 RNA and **(B)** M2 RNA crystal structures is shown. The mF_o-DF_c difference map (green mesh at +2.5 σ and red at -2.5 σ) shows a good fit of the water molecules (red colored spheres) and the U-U mismatch (stick representation) geometries in both crystal structures.

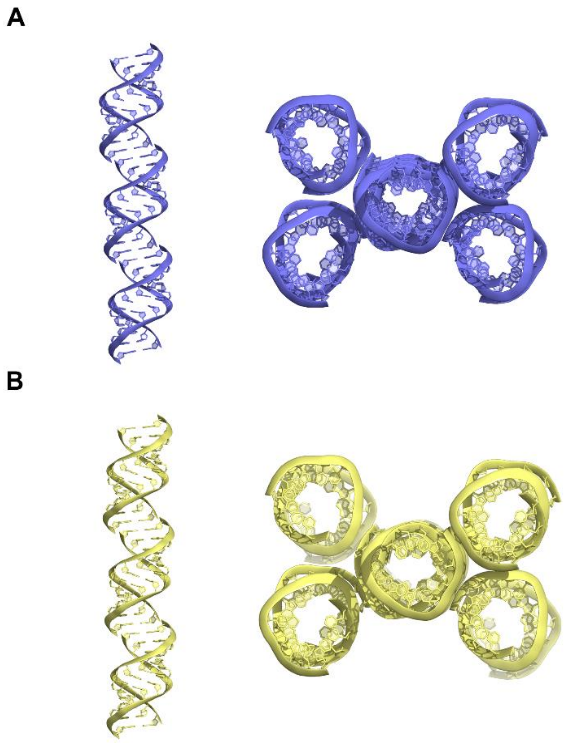

Figure S3: Crystal packing for **(A)** M3 RNA and **(B)** M2 RNA duplexes showed a typical end-to-end continuous packing in side- and top-view.

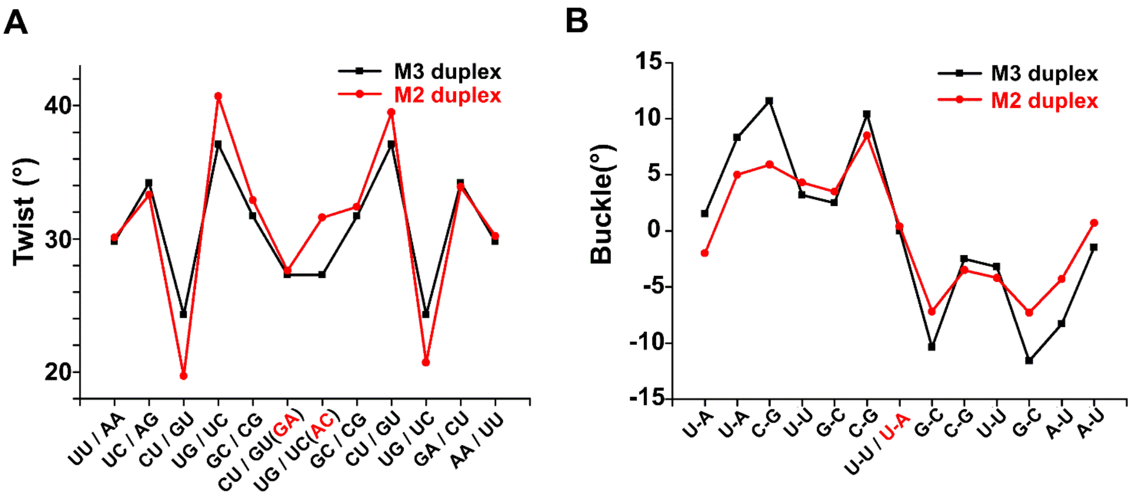

Figure S4: Significant differences observed in **(A)** Twist and **(B)** Buckle parameters for M3 RNA (black line) and M2 RNA duplexes (red line). Interestingly, the parameters for M3 RNA showed overall symmetry in duplex structure whereas, such symmetry is absent in the M2 crystal structure.

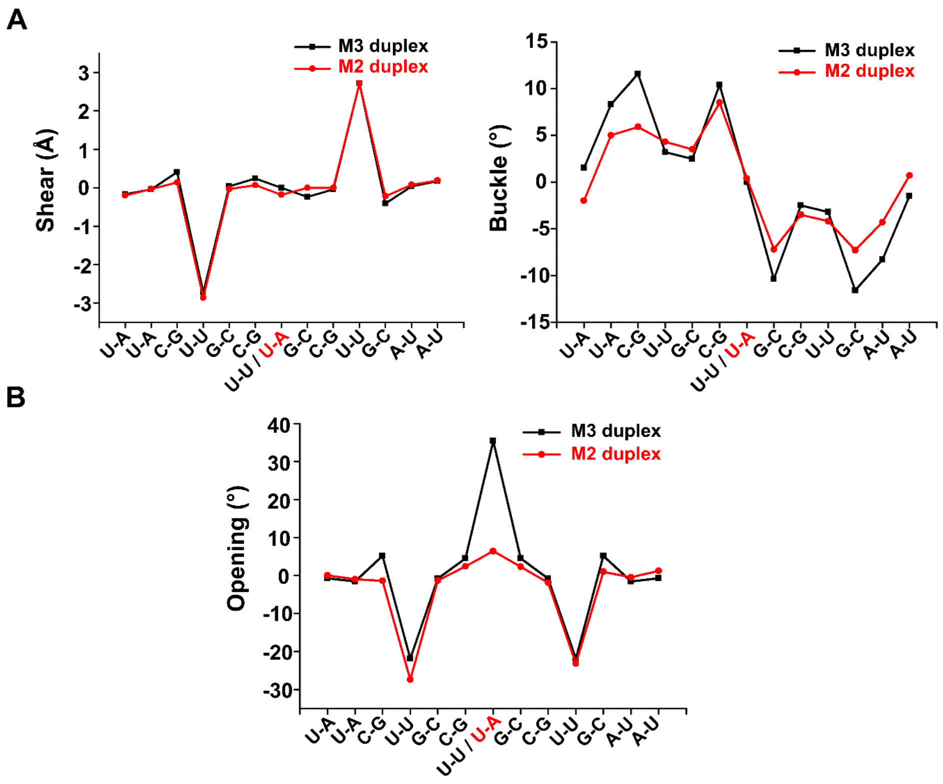

Figure S5: Significant differences observed in base-pair **(A)** Shear and Buckle and **(B)** Opening parameters for M3 RNA (black line) and M2 RNA duplexes (red line).

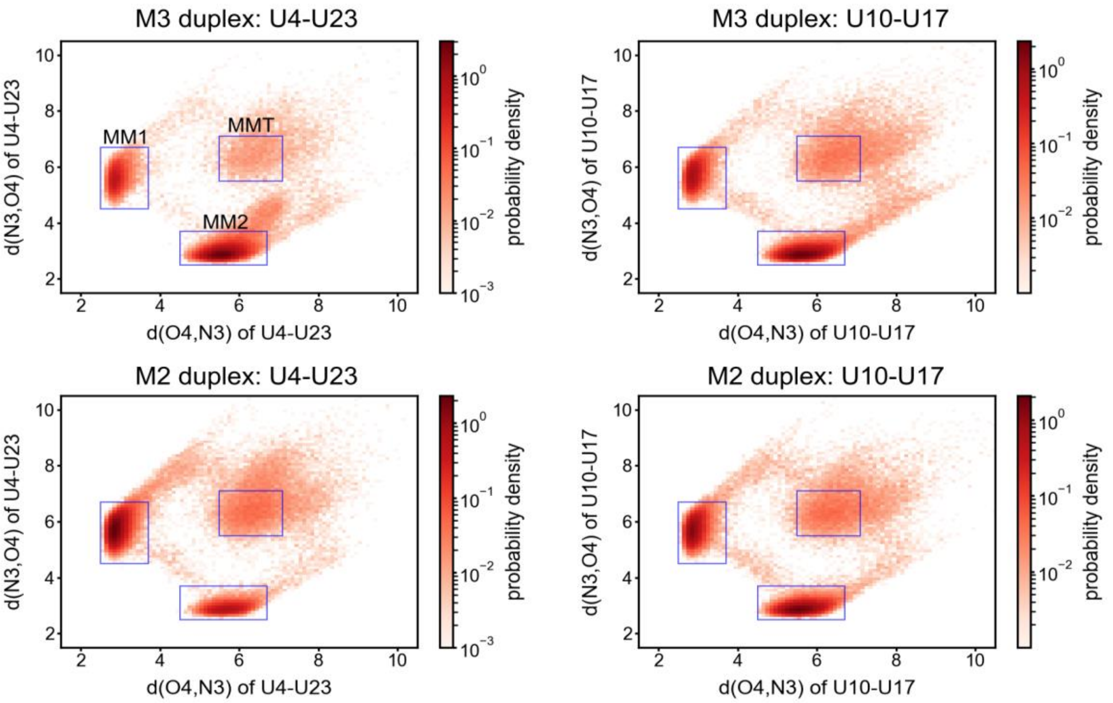

Figure S6: The joint probability density of d(O4,N3) and d(N3,O4) of U-U mismatches in the M3 duplex and the M2 duplex. The results shown here are qualitatively similar to the results presented in result section and Figure 3B.

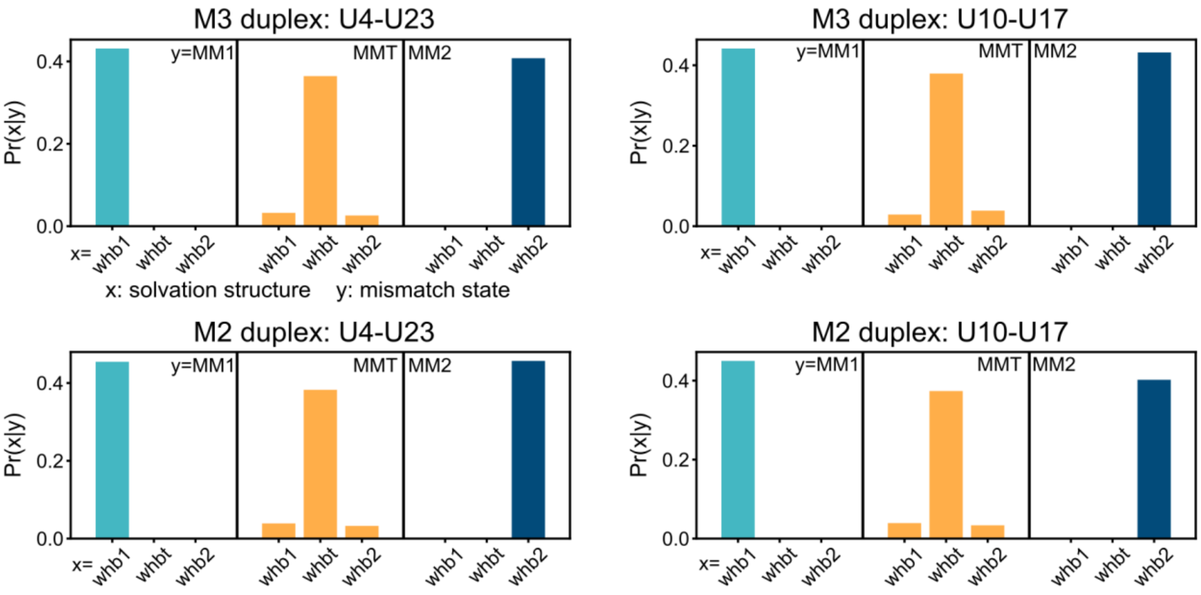

Figure S7: For U-U mismatches in the M3 duplex and the M2 duplex, the probability of finding a specific solvation structure x given a particular U-U mismatch state y. The results shown here are qualitatively similar to the results presented in Results section and Figure 3C.

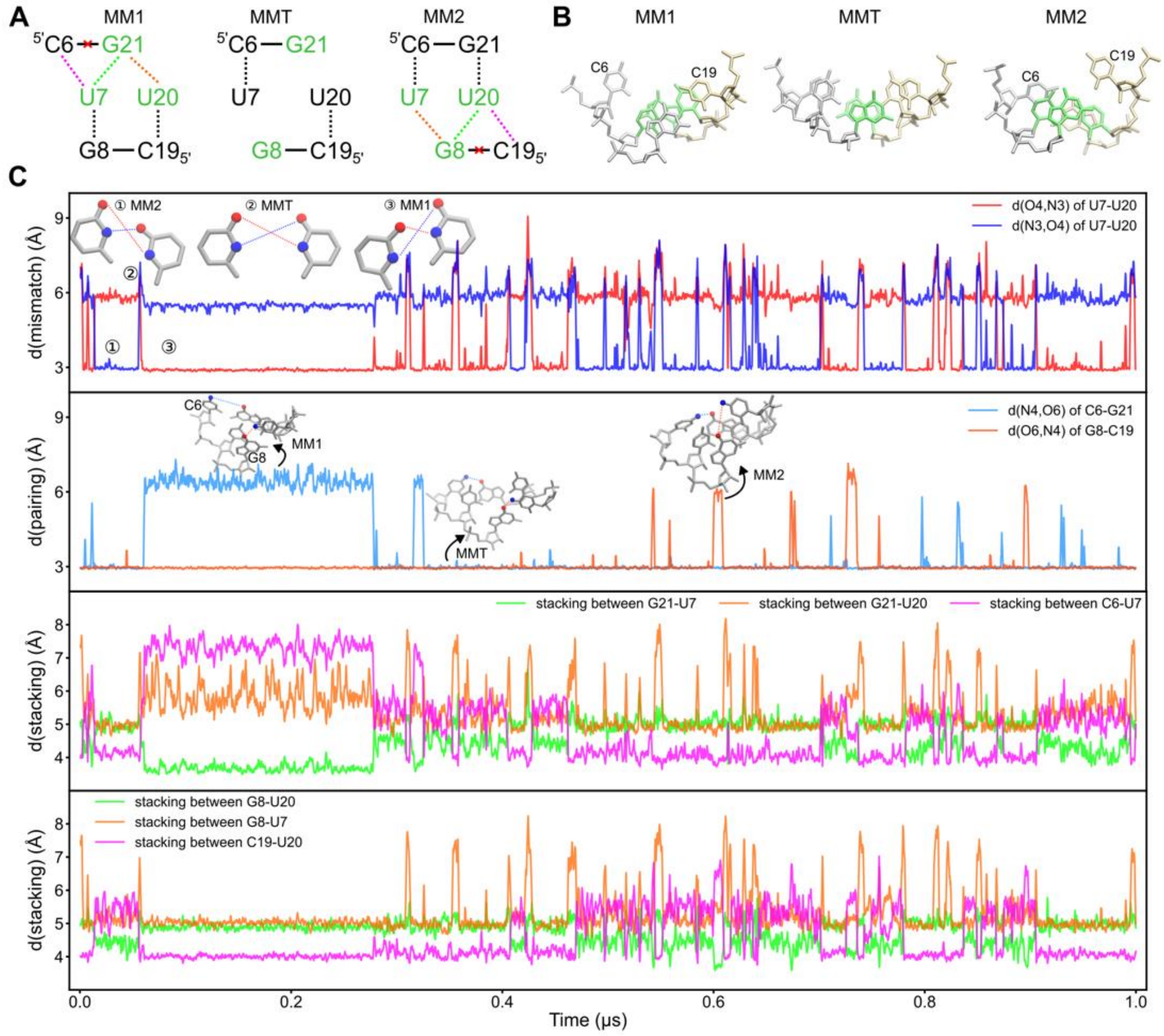

Figure S8: Disruption of adjacent C-G base pairings and cross-strand stacking interactions. **(A)** Schematic diagrams illustrating correlations among U-U mismatch states, pairing disruptions, and cross-strand stacking. Red crosses denote C-G base pairing disruptions, black dotted lines represent canonical stacking, while magenta and orange dotted lines depict disrupted stacking. Green dotted lines indicate cross-strand stacking. **(B)** Molecular graphics corresponding to schematics in (A), with perspective from bottom to top. **(C)** First panel: Time series of d(O4,N3) and d(N3,O4) in MD simulation, with insets showing transitions between MM2, MMT, and MM1. Second panel: Time series of d(N4,O6) for C6-G21 and C19-G8, illustrating adjacent C-G base pair disruptions, with insets displaying corresponding trajectory snapshots. Third and fourth panels: Time series of d(stacking), defined as the distance between the centers of six-membered rings of guanosine and uridine.

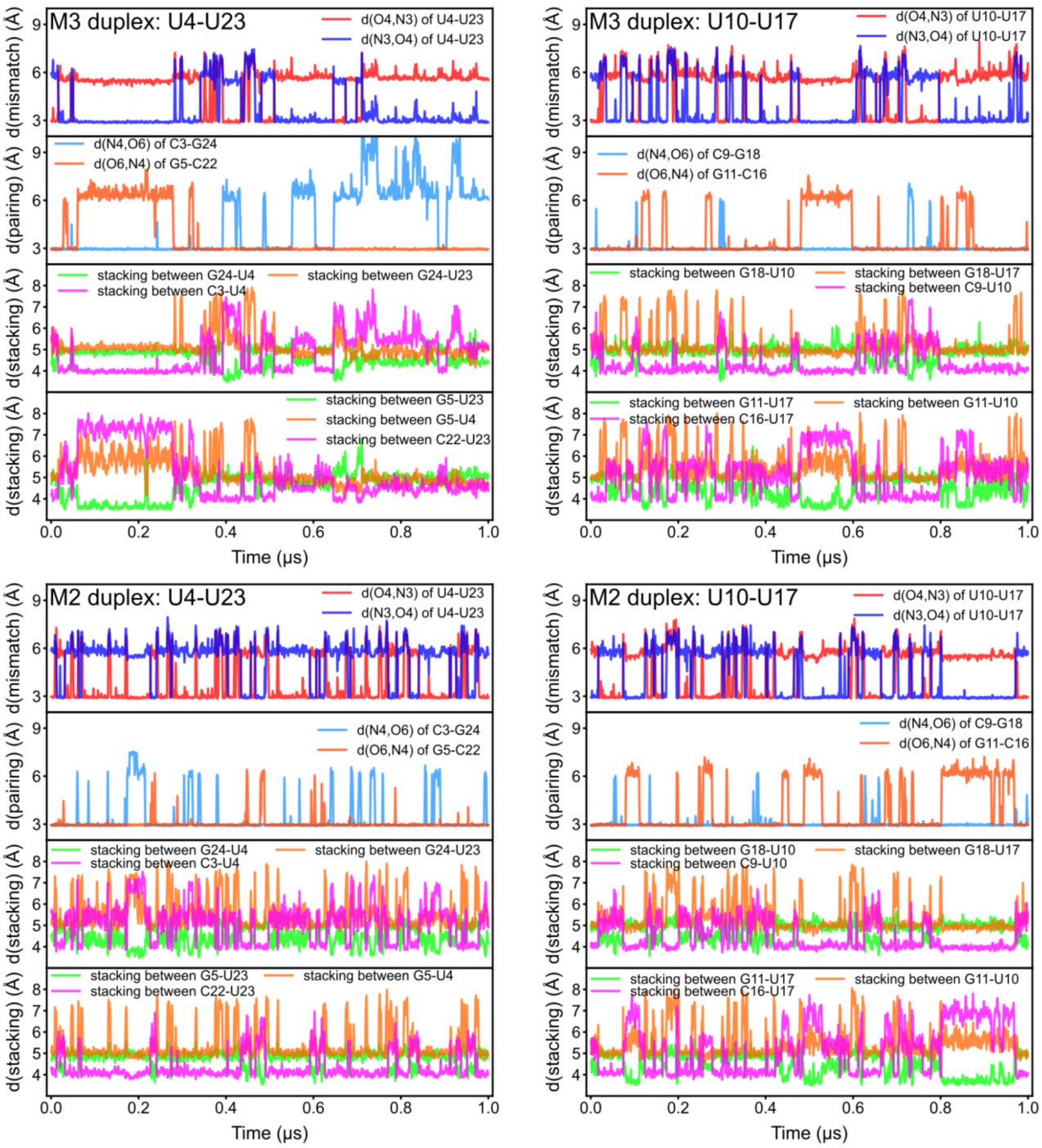

Figure S9: The time series of d(O4,N3), d(N3,O4) and d(stacking) observed in the MD simulations of U-U mismatches in the M3 duplex and the M2 duplex. The results shown here are qualitatively similar to the results presented in the supplementary note and Figure S8.

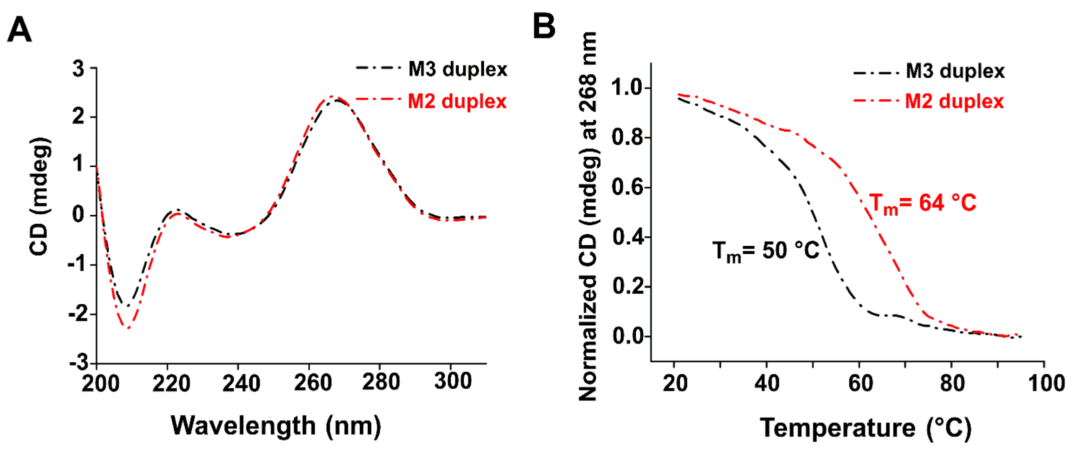

Figure S10: Circular dichroism (CD) spectra of M3 (black line) and M2 RNA duplexes (red line). **(A)** Differences in CD intensities of M3 and M2 RNA duplexes in a buffer containing 20 mM MOPS (pH 6.5) and 1 mM spermine tetrahydrochloride at 25 °C. Under identical conditions, the M2 RNA duplex enhances the band at 208 nm (negative) and 276 nm (positive), respectively. **(B)** CD Melting temperature profiles of M3 and M2 RNA duplexes in the same buffer is shown.

Supplementary tables

Table S1: The values for nucleic acid main chain and backbone torsion angles and sugar pucker types obtained for r(UUCUGCUGCUGAA)$_2$ (M3 duplex) and r(UUCUGCUGCUGAA) /r(UUCUGCAGCUGAA) (M2 duplex) using Curves plus web server.

M3 duplex								
**Strand r(UUCUGCUGCUGAA)**								
**Base**	**α**	**β**	**γ**	**δ**	**ε**	**ζ**	**χ**	**Puckering**
U-1	---	141.6	57.7	80.4	-145.9	-79.6	-160.5	*C3'-endo*
U-2	-60.4	168.4	49.3	77.5	145.6	-73.1	-159.6	*C3'-endo*
C-3	-64.8	166.0	57.1	79.2	-159.6	-88.8	-165.4	*C3'-endo*
U-4	-52.5	179.5	49.8	77.6	-145.9	-61.0	-166.8	*C3'-endo*
G-5	-64.3	-179.3	52.3	79.3	-141.8	-72.2	-169.4	*C3'-endo*
C-6	-67.4	169.0	56.7	77.3	-144.3	-68.3	-166.0	*C3'-endo*
U-7	-61.0	168.6	61.8	80.6	-163.9	-85.4	-166.0	*C3'-endo*
G-8	-56.3	179.1	50.3	78.6	-154.2	-71.1	-169.7	*C3'-endo*
C-9	-61.7	-177.0	49.7	80.8	-156.1	-67.3	-154.8	*C3'-endo*
U-10	134.4	-164.5	-160.6	82.5	-137.7	-80.2	-168.4	*C3'-endo*
G-11	-60.3	162.6	52.6	79.5	-154.7	-73.0	-168.5	*C3'-endo*
A-12	-66.8	-177.1	49.6	78.6	-150.3	-71.5	-162.3	*C3'-endo*
A-13	-62.5	167.5	64.3	82.1	---	---	-160.7	*C3'-endo*

M2 duplex								
**Strand I r(UUCUGCUGCUGAA)**								
**Base**	**α**	**β**	**γ**	**δ**	**ε**	**Z**	**χ**	**Puckering**
U-1	---	137.3	60.4	80.7	-145.3	-82.8	-167.5	*C3'-endo*
U-2	-52.6	167.7	49.0	80.6	-147.9	-75.8	-161.8	*C3'-endo*
C-3	-56.8	165.2	55.2	80.6	-153.0	-41.2	-160.8	*C3'-endo*
U-4	-146.9	117.4	159.7	81.1	-127.6	-68.4	-179.5	*C3'-endo*

G-5	-62.2	177.0	48.1	78.1	-140.3	-74.8	-169.4	*C3'-endo*
C-6	-61.3	168.1	51.5	76.5	-145.6	-68.3	-163.7	*C3'-endo*
U-7	-61.7	167.9	65.5	74.1	-156.4	-79.7	-169.6	*C3'-endo*
G-8	-60.5	175.7	53.5	80.3	-152.3	-74.1	-166.7	*C3'-endo*
C-9	-55.3	179.6	44.8	80.0	-156.3	-69.4	-156.4	*C3'-endo*
U-10	-139.9	-159.3	-169.2	81.3	-144.5	-78.5	-170.8	*C3'-endo*
G-11	-60.1	171.6	49.4	79.2	-153.4	-72.8	-166.3	*C3'-endo*
A-12	-66.6	178.7	48.4	78.0	-145.9	-75.9	-165.8	*C3'-endo*
A-13	-56.0	165.1	62.1	81.8	---	---	-163.0	*C3'-endo*

M2 duplex								
**Strand II r(UUCUGCAGCUGAA)**								
**Base**	**α**	**β**	**γ**	**δ**	**ε**	**Z**	**χ**	**Puckering**
rA-26	-57.7	162.7	65.4	81.4	---	---	-164.0	*C3'-endo*
rA-25	-68.1	179.8	49.3	78.2	-146.0	-76.4	-166.0	*C3'-endo*
rG-24	-61.2	172.4	50.2	79.3	-153.1	-72.4	-166.6	*C3'-endo*
rU-23	137.7	-157.8	-166.9	81.6	-143.4	-78.1	-170.8	*C3'-endo*
rC-22	-54.1	179.3	44.0	79.6	-155.1	-70.6	-156.6	*C3'-endo*
rG-21	-60.4	176.6	54.8	80.0	-151.7	-74.5	-167.9	*C3'-endo*
rA-20	-63.9	169.2	63.8	75.9	-157.3	-78.7	-170.0	*C3'-endo*
rC-19	-63.6	169.4	53.3	76.6	-147.0	-69.1	-164.6	*C3'-endo*
rG-18	-62.6	178.8	50.1	78.5	-142.0	-73.4	-169.8	*C3'-endo*
rU-17	-65.3	169.6	68.5	70.7	-149.1	-69.6	-169.4	*C3'-endo*
rC-16	-62.0	168.8	49.7	77.5	-154.6	-74.5	-160.6	*C3'-endo*
rU-15	-61.0	169.3	54.1	80.5	-144.5	-75.6	-163.4	*C3'-endo*
rU-14	---	146.0	59.8	79.4	-147.1	-80.8	-167.3	*C3'-endo*

Table S2: The values for nucleic acid local base-pair and base-pair steps parameters obtained for $r(UUCUGCUGCUGAA)_2$ (M3 duplex) and r(UUCUGCUGCUGAA)/(UUCUGCAGCUGAA) (M2 duplex) using Curves plus web server.

(A) Local base pair parameters

M3 duplex						
**Pair**	**Shear**	**Stretch**	**Stagger**	**Buckle**	**Propeller**	**Opening**
U-A	-0.17	-0.07	-0.04	1.5	-9.9	-0.7
U-A	-0.04	-0.11	-0.21	8.3	-5.8	-1.6
C-G	0.41	-0.06	-0.30	11.6	-9.8	5.2
U-U	-2.72	-1.12	-0.38	3.2	-11.0	-21.9
G-C	0.04	0.08	0.04	2.5	-6.5	-0.8
C-G	0.24	-0.05	-0.04	10.4	-8.9	4.5
U-U	-0.00	1.47	-0.34	0.0	-10.7	35.4
G-C	-0.24	-0.05	-0.04	-10.4	-8.9	4.5
C-G	-0.04	0.08	0.04	-2.5	-6.5	-0.8
U-U	2.72	-1.12	-0.38	-3.2	-11.0	-21.9
G-C	-0.41	-0.06	-0.30	-11.6	-9.8	5.2
A-U	0.04	-0.11	-0.21	-8.3	-5.8	-1.6
A-U	0.17	-0.07	-0.04	-1.5	-9.9	-0.7

M2 duplex						
**Pair**	**Shear**	**Stretch**	**Stagger**	**Buckle**	**Propeller**	**Opening**
U-A	-0.20	-0.09	0.09	-2.0	-6.0	-0.0
U-A	-0.03	-0.11	-0.07	5.0	-3.1	-1.0
C-G	0.14	-0.18	-0.04	5.9	-7.3	-1.4
U-U	-2.86	-1.31	-0.22	4.3	-13.3	-27.4
G-C	-0.03	-0.07	0.27	3.5	-7.4	-1.3
C-G	0.07	-0.09	-0.03	8.5	-10.3	2.4
U-A	-0.18	-0.32	0.18	0.4	-10.5	6.4
G-C	0.00	-0.12	0.05	-7.2	-11.0	2.3
C-G	0.00	-0.08	0.26	-3.5	-7.1	-1.9
U-U	2.72	-1.22	-0.16	-4.2	-12.9	-23.2
G-C	-0.22	-0.13	-0.23	-7.3	-5.6	1.0
A-U	0.08	-0.09	-0.06	-4.3	-3.0	-0.5
A-U	0.19	-0.08	0.02	0.7	-5.4	1.2

(B) Local base-pair step parameters

M3 duplex						
**Step**	**Shift**	**Slide**	**Rise**	**Tilt**	**Roll**	**Twist**
UU/AA	-0.51	-1.40	3.16	-1.5	7.2	29.8
UC/AG	0.79	-1.43	3.18	0.3	6.9	34.2
CU/GU	-1.90	-1.88	3.47	-3.3	8.4	24.3
UG/UC	0.28	-2.14	3.39	-7.5	7.2	37.1
GC/CG	0.55	-1.77	3.10	-0.2	3.2	31.7
CU/GU	2.45	-2.12	3.43	5.2	4.9	27.3
UG/UC	-2.45	-2.12	3.43	-5.2	4.9	27.3
GC/CG	-0.55	-1.77	3.10	0.2	3.2	31.7
CU/GU	-0.28	-2.14	3.39	7.5	7.2	37.1
UG/UC	1.90	-1.88	3.47	3.3	8.4	24.3
GA/CU	-0.79	-1.43	3.18	-0.3	6.9	34.2
AA/UU	0.51	-1.40	3.16	1.5	7.2	29.8
UU/AA	-0.51	-1.40	3.16	-1.5	7.2	29.8

M2 duplex						
**Step**	**Shift**	**Slide**	**Rise**	**Tilt**	**Roll**	**Twist**
UU/AA	-0.60	-1.72	3.08	-1.9	6.7	30.1
UC/AG	0.30	-1.46	3.31	0.1	4.5	33.3
CU/GU	-0.86	-2.37	3.27	1.0	7.9	19.7
UG/UC	0.33	-2.06	3.32	-8.3	7.2	40.7
GC/CG	0.49	-1.66	3.11	2.2	4.8	32.9
CU/GA	1.07	-2.27	3.37	0.4	6.7	27.6
UG/AC	-0.90	-1.99	3.38	-0.8	5.2	31.6
GC/CG	-0.50	-1.71	3.16	-1.6	5..3	32.4
CU/GU	-0.14	-2.00	3.28	8.1	7.7	39.5
UG/UC	0.87	-2.30	3.37	0.5	6.0	20.7
GA/CU	-0.26	-1.40	3.24	-0.7	6.2	33.9
AA/UU	0.65	-1.70	3.13	2.5	7.2	30.2
UU/AA	-0.60	-1.72	3.08	-1.9	6.7	30.1

Supplementary movies

Movie S1: The transition from MM1 to MM2 states of the U7-U20 mismatch in the M3 duplex. This movie showcases the surrounding water molecules to illustrate the solvation around U-U mismatch. Notably, during the transition, water molecules pass through the center of the U-U mismatch. The movie covers the simulation time from 613 ns to 617 ns.

Movie S2: The transition from MM2 to MM1 states of the U7-U20 mismatch in the M3 duplex. This movie displays water molecules surrounding the U7-U20 mismatch to illustrate the solvation around U-U mismatch. During the transition, water molecules pass through the center of the U-U mismatch. The movie covers from 55 ns to 59 ns of the simulation.

Movie S3: Concurrent disruption of adjacent C6-G21 base pairings and cross-strand stacking between U7 and G21. When the U7-U20 mismatch is in the MM1 state, the movie reveals concurrent C6-G21 pairing disruption and cross-strand stacking. Note that the left-bottom cytosine is C6 and the right-bottom guanosine is G21. The movie covers the simulation time from 0 ns to 400 ns.

Movie S4: Concurrent disruption of adjacent G8-C19 base pairings and cross-strand stacking interaction between U20 and G8 during the MM2 state of the U7-U20 mismatch. The movie highlights the concurrent G8-C19 pairing disruption and cross-strand stacking. Additionally, when U7-U20 is in the MMT intermediate state, the middle space between the U7-U20 mismatch enlarges (due to a bridging water molecule pushing the uridines outward, although water molecules are not shown for clarity), resulting in well-maintained canonical stacking interactions U7→C6 and U20→C19. Observe that the left-top guanosine is G8 and the right-top cytosine is C19. The movie covers the simulation time from 500 ns to 800 ns.
